# Machine‐Learning–Based Prediction of Hypertension and Its Risk Factors Among Adults in the Northern Region of Bangladesh

**DOI:** 10.1155/jdr/1799434

**Published:** 2026-03-20

**Authors:** Most. Nusrat Jahan Resma, Md. Kabir Bin Karim, Isteaq Kabir Sifat, Md. Abdul Kayum, Md. Kaderi Kibria

**Affiliations:** ^1^ Department of Statistics, Hajee Mohammad Danesh Science and Technology University, Dinajpur, Bangladesh, hstu.ac.bd; ^2^ Department of Electronics and Communication Engineering, Hajee Mohammad Danesh Science and Technology University, Dinajpur, Bangladesh, hstu.ac.bd; ^3^ Department of Statistics, University of Rajshahi, Rajshahi, Bangladesh, ru.ac.bd

**Keywords:** Bangladesh, Dinajpur District, hypertension, machine-learning technique, risk factor, SHAP analysis

## Abstract

Hypertension (HTN) is a major contributor to cardiovascular morbidity and mortality globally. Its burden is rising in low‐ and middle‐income countries like Bangladesh. Despite its rising prevalence, predictive modeling of HTN using advanced analytical approaches remains limited in rural populations. This study is aimed at predicting HTN and identifying its key risk factors among adults in Dinajpur District, Bangladesh, using machine‐learning (ML) techniques. A community–based cross‐sectional study was conducted among 1026 adults aged ≥ 30 years between December 2024 and February 2025. Data on sociodemographic, behavioral, and clinical characteristics were collected through a structured questionnaire. Feature selection was performed using recursive feature elimination (RFE), Boruta‐based feature selection (BFS), and random forest (RF) methods. Five ML algorithms such as logistic regression, decision tree, RF, extreme gradient boosting, and light gradient boosting machine were trained and evaluated based on accuracy, precision, recall, F1 score, and area under the curve (AUC). SHAP (SHapley Additive exPlanations) analysis was employed to interpret model outputs. The prevalence of HTN among participants was 36.5%. The RF model achieved the highest predictive performance with 72% accuracy, 71% precision, 72% recall, 71% F1 score, and an AUC of 0.80. Thirteen significant predictors were identified, with age, body weight, sweets consumption, vigorous activity, education, family size, height, and family income emerging as the most influential determinants. These findings demonstrate the potential of ML models in predicting HTN and identifying modifiable risk factors. The results provide actionable insights to support early detection, targeted interventions, and effective resource allocation for HTN prevention and control in rural Bangladesh.

## 1. Introduction

Hypertension (HTN), or high blood pressure, is a leading global health concern and a major risk factor for cardiovascular diseases (CVDs), stroke, kidney failure, and premature mortality [1–3]. Globally, over 1.28 billion adults worldwide are affected, men and women experiencing prevalence rates of approximately one in four and one in five, respectively [4–6]. The condition significantly increases the risk of heart disease, peripheral vascular disease, and stroke, contributing to substantial morbidity, mortality, and economic burden due to healthcare costs and lost productivity [7, 8]. By 2025, the number of adults aged 30–79 living with HTN is projected to reach 1.56 billion, with nearly two‐thirds residing in low‐ and middle‐income countries (LMICs) [9]. These trends highlight the urgent need for early detection and prevention strategies to mitigate the health and economic impacts of HTN.

In Bangladesh, the prevalence of HTN has risen significantly in recent years, driven by urbanization, sedentary lifestyles, unhealthy diets, tobacco use, and rising obesity rates [10, 11]. The burden is particularly pronounced in rural and semiurban areas where limited health literacy and poor access to healthcare services prevail. Low awareness of healthy lifestyle practices further exacerbates the situation [12]. Dinajpur is a rural district in northern Bangladesh that exemplifies these challenges. However, no comprehensive community‐based study has examined the prevalence and determinants of HTN using advanced predictive methods. Accurate prediction of HTN and identification of key risk factors are critical for enabling timely interventions. Conventional statistical models such as logistic regression (LR) and Cox proportional hazards models have been widely used to study HTN, but they often fail to capture complex, nonlinear interactions among multiple predictors [13–15]. Machine‐learning techniques, including random forest (RF), support vector machine (SVM), and gradient boosting (GBoost) methods offer superior predictive performance and the ability to uncover influential risk factors from multidimensional datasets [16–19]. For instance, a study across three South Asian countries applied six ML classifiers (decision tree [DT], RF, GBM, extreme gradient boosting [XGBoost], LR, and LDA) to predict HTN, with XGBoost achieving the highest accuracy (90%), followed by RF (89%) and DT (83%) [20]. Another study reported that RF achieved the highest accuracy (80.12%) and an AUC of 85.96%, outperforming KNN, DT, and NB [16]. Using data from the Bangladesh Demographic and Health Survey 2017–18, GBoost model achieved the best performance (accuracy 66.98%, recall 97.92%, F1‐score 78.99%, and AUC 0.669) among 6965 individuals aged ≥ 35 years [21]. Similarly, another study using medical data from Bangladesh reported that the RF algorithm demonstrated the highest accuracy (73.86%) in predicting HTN among high‐risk adults [22]. Leveraging machine‐learning approaches can provide actionable insights for early detection and targeted public health interventions. This study is aimed at predicting HTN and identifying its associated risk factors among adults in Dinajpur District, Bangladesh using advanced algorithms. The application of these techniques seeks to improve predictive accuracy and guide evidence‐based strategies for prevention and control in rural populations.

## 2. Materials and Methods

### 2.1. Study Design, Setting, and Population

A community–based cross‐sectional study was conducted among adults aged ≥ 30 years in Dinajpur District, which is a rural region in northern Bangladesh. The study was conducted from December 10, 2024, to February 10, 2025. The area was selected due to limited healthcare access, low health literacy, and a potentially high risk of undiagnosed HTN. Adults aged ≥ 30 years who had their blood pressure measured at certified diagnostic centers within the preceding month were included in this study. Participants aged ≥ 30 years were selected because HTN prevalence rises markedly after early adulthood, making this age group more appropriate for identifying meaningful risk factor patterns [23–25]. We excluded those for whom recent blood pressure measurement was not available (i.e., not measured at an accredited diagnostic center in the past month) and those with incomplete or missing blood pressure records. In addition, subjects with inconclusive health information were excluded. Previous diagnosis of HTN was not considered an exclusion criterion, as our aim was to measure both previously diagnosed and undiagnosed HTN among adults. A multistage sampling technique was employed to select study participants from Dinajpur District. In the first stage, four upazilas named Dinajpur Sadar, Birganj, Parbatipur, and Nawabganj were randomly selected. In the second stage, three unions were randomly chosen from each selected upazila. In the final stage, households within each selected union were identified using systematic random sampling, and one eligible adult (aged ≥ 30 years) was randomly selected from each household for a face‐to‐face interview. Random selection at each stage ensured representativeness of the adult population. Sampling weights were not applied as the study focused on predictive modeling rather than population‐level inference. The minimum sample size calculated via Cochran′s formula [26] was 384, but data were collected from 1026 participants to improve representativeness (see Figure S1).

### 2.2. Questionnaire, Data Collection, and Outcome Measurement

A structured questionnaire was developed in English based on a literature review related to HTN [9, 27–29] and subsequently translated into Bengali. It was validated through a pilot test on 50 participants from outside the study area. Content validity was ensured and internal consistency (Cronbach^′^s alpha = 0.79) confirmed reliability. It captured sociodemographics, behavioral factors, medical history, comorbidities, physical measurements, and HTN treatment/control. HTN was defined as systolic BP ≥ 140 mmHg and/or diastolic BP ≥ 90 mmHg [4, 5], coded as 1 (hypertensive) or 0 (nonhypertensive). Trained enumerators conducted face‐to‐face interviews, measured height and weight, and ensured data quality and ethical compliance.

### 2.3. Predictor Variables

A total of 34 predictor variables were included in this study, which were selected based on prior research on HTN [28, 30–32] (see Table S1 for details). These variables encompassed sociodemographic, lifestyle, anthropometric, and medical history factors. Sociodemographic variables included residence (rural/urban), gender (male/female), age, marital status (single, married, divorced/widowed), education level (no education to honors/diploma or above), religion (Muslim, Hindu, Buddhist), family income, family size, occupation (farmer, business, day labor, driver, employee, housewife, retired, others, unemployment), housing ownership (personal/rental), and loan status (yes/no). Lifestyle‐related variables covered smoking status (ever smoked or not), betel leaf/gul consumption, tea/coffee intake, dietary habits (frequency of fruit, vegetable, meat, and sweet consumption), added salt usage, physical activity (vigorous activity, sports participation, walking), and transportation mode (foot, bicycle, engine vehicle). Medical and health‐related variables included self‐reported HTN status, HTN measurement, family history of HTN, diabetes status, family history of diabetes, kidney disease, CVD, and family history of CVD. Anthropometric measures included height, weight, and body mass index (BMI), categorized as normal, overweight, or obese.

### 2.4. Data Processing and Scaling

Data was coded in Excel and imported into SPSS (v26) for cleaning and preprocessing. Missing values were imputed using the median for numerical variables and the mode for categorical variables [33]. This approach was selected because the proportion of missing data was low, and the sample size was limited [34]. The selected imputation strategy was appropriate for preserving the original data structure while minimizing the risk of introducing additional variability. Outliers were treated using interquartile range (IQR) methods [35]. Multicollinearity was assessed using variance inflation factor (VIF > 10 considered high) [36]. After completing data cleaning and transformation procedures, the dataset was prepared for subsequent analysis and model development. To ensure uniform contribution of features and optimize model performance [37], min–max scaling [38] was applied to all numerical variables, transforming them to a range between 0 and 1 using the formula:

Xscaled=X−XminXmax−Xmin

This normalization step helped prevent any single feature from disproportionately influencing the machine‐learning models.

### 2.5. Feature Selection

The HTN dataset included 34 candidate predictors including both categorical and numerical variables. Although the dimensionality of the dataset was not large, incorporating all predictors without selection could introduce noise and negatively impact model performance. To ensure robust and stable feature identification, three complementary feature selection approaches were applied, such as RFE, BFS, and RF. These methods were chosen because they rely on different underlying principles. RFE is a wrapper‐based technique that iteratively removes less informative variables based on model performance [33, 39], RF importance captures nonlinear relationships and interactions between predictors [37, 40], and Boruta provides a statistically grounded comparison of real features against randomized shadow features [37, 41, 42]. Using these methods in parallel allowed us to reduce method‐specific bias and identify predictors that consistently demonstrated importance across multiple selection frameworks. Only features consistently identified by all three methods were retained to enhance model interpretability and reduce the risk of overfitting [19, 41].

Common features=∩irSelected features from HTN dataset

where *r* is the number of feature selection techniques (here *r* = 3).

### 2.6. Machine‐Learning Algorithms and Evaluation

Five ML algorithms such as LR, DT, RF, Extreme Gradient Boosting (XGBoost), and Light Gradient Boosting Machine (LightGBM) were employed to predict HTN (see Appendix S1). LR was selected as a baseline model for binary classification due to its simplicity, interpretability, and widespread use in epidemiological studies for identifying associations between predictors and a binary outcome [1]. DT was applied as a nonparametric, tree‐based model capable of capturing nonlinear relationships and interactions among predictors while providing clear visual interpretability for clinical insights [43]. RF, an ensemble of decision trees, was used to enhance predictive robustness and reduce overfitting, leveraging bagging techniques to improve generalization across heterogeneous datasets [44]. XGBoost and LightGBM were employed to handle complex, high‐dimensional data efficiently, offering superior accuracy and computational speed. These gradient boosting algorithms iteratively improve model performance by minimizing prediction errors and are particularly effective for structured medical data with nonlinear patterns [20]. Model performance was evaluated using a confusion matrix to derive accuracy, precision, recall (sensitivity), F1‐score, specificity, and error rate, whereas the area under the receiver operating characteristic curve (AUC‐ROC) assessed classification performance across varying thresholds.

### 2.7. Data Partition

The dataset of 1026 observations was divided into training and testing subsets using stratified random selection to retain the original class distribution. Here, 70% of the data (*n* = 718) was allocated to the training set, with the remaining 30% (*n* ≈ 308) reserved for model evaluation. Stratification guaranteed that the proportion of hypertensive (36.5%) and nonhypertensive (63.5%) patients remained constant in both subsets, reducing sampling bias and retaining the target variable′s representativeness across partitions.

### 2.8. Cross‐Validation and Hyperparameter Tuning

The above‐mentioned ML algorithms have additional parameters known as hyperparameters. To improve the model′s performance, the user can specify hyperparameters before the learning process. The hyperparameter values in the training set were adjusted by the grid search technique using a repeating tenfold (K10) cross‐validation procedure. The K10 approach involves separating a training subset and verification set from the training dataset in a 7:3 ratio. Details of parameter ranges are provided in Table S2. Data analysis was conducted using IBM SPSS Statistics (Version 31) for descriptive statistics, whereas R (Version 4.5.0) and Python (Version 3.12.0) were used for ML‐based prediction and data visualization. The overall workflow of this study is shown in Figure S2.

## 3. Results

### 3.1. Demographic Profiles of the Respondents

A total of 1026 adults aged ≥ 30 years participated in the study. Among them, the majority residing in rural areas (78.5%) (see Table 1 for details). Most participants were married (89.0%) and 51.0% were male. Age distribution showed that 34.1% were between 30 and 39 years, followed by 28.3% who were 40–49 years, and 19.6% who were 50–59 years. Educational attainment varied, where 26.1% were illiterate and 16.3% had higher education. The most common occupation of participants was housewife (44.2%), followed by employed individuals (12.0%), agriculture (10.5%), day labor (10.1%), and shopkeepers (5.8%). Monthly income was below 20,000 BDT for 44.2% of participants, whereas only 5.6% earned above 55,000 BDT. Most respondents (96.5%) reported owning their own house. Regarding BMI, 63.9% had normal weight, 30.0% were overweight, and 6.0% were obese. Additionally, 59.0% of respondents reported having existing loans, indicating a considerable level of financial burden within the population.

**Table 1:** Demographic characteristics of study respondents (*n* = 1026).

Variable	Categories	Frequency (%)	Variable	Categories	Frequency (%)
Residence	Urban	221 (21.5%)	Occupation	Agriculture	108 (10.5%)
	Rural	805 (78.5%)		Business	92 (9.0%)
Gender	Male	523 (51.0%)		Day labor	104 (10.1%)
	Female	503 (49.0%)		Employed	123 (12.0%)
Age	30–39	349 (34.1%)		Housewife	454 (44.2%)
	40–49	291 (28.3%)		Driver	32 (3.1%)
	50–59	201 (19.6%)		Retired	50 (4.9%)
	60–69	128 (12.4%)		Shopkeeper	59 (5.8%)
	≥ 70	57 (5.6%)		Unemployment	17 (1.7%)
Marital status	Single	29 (2.8%)	Income	< 20,000	453 (44.2%)
	Married	913 (89.0%)		20,001–35,000	367 (35.8%)
	Divorced/widowed	84 (8.2%)		35,001–55,000	149 (14.5%)
Education	No education	268 (26.1%)		55,001–80,000	40 (3.9%)
	Primary education	231 (22.5%)		> 80,000	17 (1.7%)
	Secondary education	261 (25.4%)	House owner	Personal	990 (96.5%)
	Higher secondary	99 (9.6%)		Rental	36 (3.5%)
	Honors/diploma or above	167 (16.3%)	BMI	Normal	656 (63.9%)
Loan	Yes	605 (59.0%)		Overweight	308 (30.0%)
	No	421 (41.0%)		Obese	62 (6.0%)

### 3.2. Behavioral Characteristics

**Figure 1:**
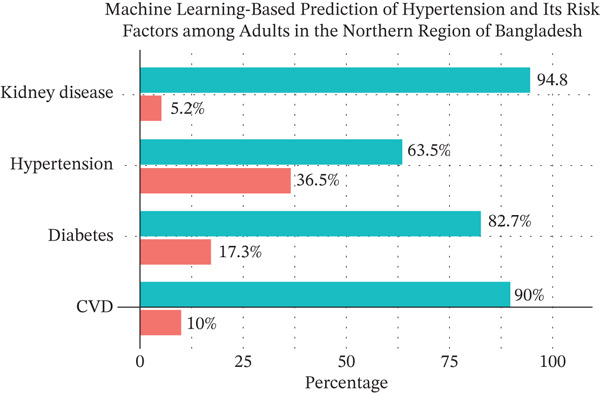
Distribution of self‐reported chronic conditions among adults.

Among the 1026 participants, 20.2% reported having ever smoked, and 31.3% consumed betel leaf or gul. Tea/coffee consumption was common (58.0%), and 52.6% frequently consumed sweets. Meat intake was moderate, with 69.7% eating it 1–2 days/week, 3.7% almost daily, and 6.0% not at all. Fruit consumption was generally low, such as 53.7% ate fruits 1–2 days/week, 24.1% for 3–4 days, 11.7% regularly (5–7 days), and 10.5% reported no fruit intake. In contrast, vegetable consumption was high, with 82.9% consuming vegetables 6–7 days per week and only 2.2% fewer than 3 days. Regarding transportation, 71.9% used motorized vehicles, 21.3% walked, and 6.8% used bicycles. Added salt during meals was reported by 30.5% of participants (see Table S3). As shown in Figure 1, HTN was the most prevalent self‐reported condition (36.5%), followed by diabetes (17.3%), CVD (10.0%), and kidney disease (5.2%), whereas the majority reported no history of these conditions.

### 3.3. Risk Factors Identification

Feature selection enhances model performance by isolating the most relevant variables associated with HTN. This study applied three methods, RFE, BFS, and RF, to select key features. Features appearing in at least two approaches were shortlisted, yielding 13 key features. These represent a mix of sociodemographic, behavioral, and anthropometric factors. The identified features comprised age, an established risk factor reflecting increasing vulnerability with advancing years; education and family income representing socioeconomic status and health literacy; family size, which may influence lifestyle and dietary practices; and occupation reflecting occupational stress and physical activity levels. Lifestyle‐related variables included ever smoking cigarettes, fruit intake, meat intake, sweet consumption, and engagement in vigorous activity, which collectively capture behavioral influences on HTN risk. Anthropometric measures such as height, weight, and BMI were also consistently selected, highlighting the contribution of body composition and obesity to HTN. Together, these 13 features provide a comprehensive profile of the demographic, socioeconomic, behavioral, and physiological determinants of HTN (see Table 2).

**Table 2:** Key risk factors for hypertension were identified by three feature selection techniques.

Variables	Data type	Description	Categorization
Age	Continuous	Respondent′s age in years	—
Education	Ordinal	Respondent′s education level	No education
			Primary education
			Secondary education
			Higher secondary
			Honors/diploma or above
Family income	Ordinal	Respondent′s family income	Less than 20,000
			20,001–35,000
			35,001–55,000
			55,000–80,000
			More than 80,000
Family size	Continuous	Number of family size	—
Occupation	Nominal	Respondent′s occupation	Farmer
			Business
			Day labor
			Driver
			Employee
			Housewife
			Retired
			Others
			Unemployment
Ever smoke cigarette	Nominal	Respondent′s ever smoke cigarette or not	Yes
			No
Eat fruit	Ordinal	Weekly frequency of fruit consumption by respondent	1–2 days
			3–4 days
			5–7 days
			None
Eat meat	Ordinal	Weekly frequency of meat consumption by respondent	1–2 days
			3–5 days
			6–7 days
			None
Eat sweet	Nominal	Whether the respondent eats sweet weekly or not	Yes
			No
Vigorous activity	Nominal	Whether the respondent engages in vigorous activity per week or not	Yes
			No
Height (inch)	Continuous	Respondent′s height in inch	—
Weight (kg)	Continuous	Respondent′s weight in kg	—
BMI	Ordinal	Body mass index level of respondents	Normal
			Overweight
			Obese

### 3.4. HTN Prediction and Model Performance

The predictive performance of the five ML models for HTN evaluated using the 13 selected features is comprehensively summarized in Table 3. Among them, the RF model demonstrated the best overall performance that achieved the highest accuracy (72%), recall (72%), F1‐score (71%), and AUC (0.80), indicating superior discriminative ability. The XGBoost model ranked second‐highest in accuracy (71%) with an AUC of 0.78, showing competitive performance compared with RF. LR and LightGBM achieved comparable results, each with 70% accuracy and an AUC of 0.73, suggesting moderate predictive capacity. In contrast, the DT model performed the weakest across all metrics, with an accuracy of 68% and the lowest AUC (0.72). Pairwise comparisons of AUCs using the DeLong test further supported these findings. The RF model achieved significantly higher AUC values than LR (*p* value = 0.001), DT (*p* value = 0.023), and LightGBM (*p* value = 0.042). However, the difference in AUC between RF and XGBoost was not statistically significant (*p* value = 0.084), which is indicating comparable discriminative performance between these two ensemble‐based models. The ROC analysis (Figure S3) further corroborates the superior predictive ability of RF that is highlighting its robustness and reliability in capturing complex patterns associated with HTN.

**Table 3:** Comparative performance metrics of machine‐learning models for hypertension prediction.

Models	Accuracy	Precision	Recall	F1‐score	AUC	*p*
LR	70%	71%	70%	70%	0.73	0.001
DT	68%	68%	68%	68%	0.72	0.023
RF	**72%**	**71%**	**72%**	**71%**	**0.80**	**Reference**
XGBoost	71%	70%	69%	70%	0.78	0.084
LightGBM	70%	70%	70%	70%	0.73	0.042

### 3.5. Interpretable Risk Factors of HTN

**Figure 2:**
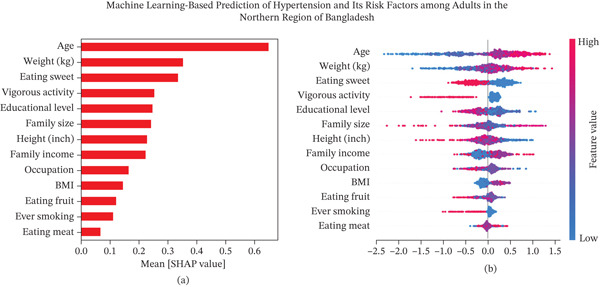
Feature importance of risk factors based on SHAP values (a) mean absolute SHAP values (b) local explanation summary.

The relative importance and directional impact of individual features on model predictions were assessed using the SHAP summary plot (Figure 2). Features are ranked in descending order based on their mean absolute SHAP values that reflect their overall contribution to the model output. Among all predictors, age emerged as the most influential factor. Higher age values depicted in red color were strongly associated with increased SHAP values, which indicated a higher predicted risk. Similarly, body weight and frequency of sweet consumption were positively associated with the HTN risk that is suggesting that higher values in these variables substantially contribute to increased predicted risk. Educational level, family size, and height also exhibited moderate effects, which indicated that lower education and larger family size tended to increase predicted risk. Socioeconomic variables such as family income and occupation demonstrated a modest but noticeable impact on the model. Lifestyle‐related features such as BMI, fruit consumption, smoking status, and meat consumption had relatively lower contributions. Overall, the SHAP analysis reveals that a combination of demographic, lifestyle, and socioeconomic factors particularly age, body weight, dietary habits, and physical activity significantly influence the model′s predictions as key modifiable risk factors.

## 4. Discussion

This study applied several ML models to predict HTN and identify its key risk factors among adults in the northern region of Bangladesh. The prevalence of HTN among adults was 36.5%, which indicates a substantial public health burden. This rate is notably higher than the national average of 27.5% reported in the BDHS 2017–18 [20] and 24.4% reported in the BHDS 2011 [45], suggesting potential regional disparities that may be shaped by socioeconomic, behavioral, and lifestyle factors. A total of 1026 individuals were recruited, and 34 potential predictors were considered. Feature selection techniques identified 13 key predictors that were used to train five ML models. Based on performance metrics, the RF model was determined to be the most suitable classifier, achieving the highest accuracy (72%) and AUC (0.80). XGBoost ranked second with an accuracy of 71% and an AUC of 0.78. Although the AUC difference between RF and XGBoost was not statistically significant (*p* = 0.084), RF showed slightly higher overall performance across multiple metrics, supporting its selection as the primary predictive model. LR and LightGBM demonstrated comparable moderate predictive performance (AUC 0.73), whereas DT performed the weakest (AUC 0.72). These results are broadly consistent with previous ML studies, but notable differences exist. For instance, a study among 6965 Bangladeshi adults aged ≥ 35 years applied four ML models and found XGBoost to be the top performer with an AUC of 0.669 and recall of 97.9% [21]. Another study in Indonesia using DT, RF, XGBoost, and LR reported that LR was the best‐performing model with an AUC of 0.829 [46]. Large‐scale analyses across Bangladesh, Nepal, and India (*n* = 818,603) indicated XGBoost outperformed other algorithms [20]. In Malaysia (*n* = 2,461), LightGBM achieved the highest accuracy (74.39%) after correlation‐based feature selection and SMOTE adjustment [47]. In the United States, ANN models yielded the highest performance in HTN prediction without explicit feature selection [48], whereas in Qatar (*n* = 987), RF demonstrated superior predictive ability compared with LR and DT [1]. Collectively, these studies highlight that ensemble‐based models, particularly RF and XGBoost, generally outperform classical models, which aligns with our findings. The slightly lower AUC observed in our study compared with some national and international datasets [19, 46, 47] may be attributed to the smaller, region‐specific sample, reliance on community‐level survey data without clinical or biochemical biomarkers, and higher homogeneity of the rural population. Despite this, the RF model′s performance remains competitive and demonstrates robust discriminative ability within the study population, underscoring its potential for local implementation. These observations emphasize the importance of contextual dataset characteristics when interpreting ML model performance and the need for external validation to enhance generalizability to other Bangladeshi regions.

SHAP analysis provided insight into the most influential predictors of HTN in our cohort. Age, body weight, sweets consumption, low physical activity, education, family size, height, and family income were identified as major contributors. These findings are consistent with prior studies linking excess weight, obesity, and sedentary behavior to higher cardiovascular risk [49, 50]. For instance, a study in China identified BMI, age, waist circumference, and family history as key HTN predictors [17], whereas a study in the United States reported poor dietary habits, and BMI significantly increased HTN risk among 7073 participants [51]. Our results further corroborate evidence that lower socioeconomic status is associated with limited healthcare access, poor diet, and increased psychosocial stress, contributing to higher HTN prevalence [52]. Dietary patterns and health literacy further interact with socioeconomic factors. For instance, low education and income may limit knowledge of HTN risk and access to preventive measures, whereas larger family size may increase stress and reduce opportunities for healthy behaviors [53]. Other lifestyle factors, including tobacco and alcohol use, overweight/obesity, and abdominal obesity, were consistent with international evidence linking these behaviors to elevated HTN risk [7, 54]. Modifiable behaviors such as physical activity and diet emerged as actionable targets, whereas nonmodifiable factors like age and family history provide critical context for risk stratification. Our findings align with studies from South Korea [55], Turkey [56], and Canada [57], demonstrating that simple demographic and lifestyle predictors can achieve strong predictive performance and are useful in real‐world applications. Higher mean systolic and diastolic blood pressure, male sex, older age, marital status, higher socioeconomic status, illiteracy, and retirement were also linked to HTN according to a cross‐sectional study conducted in urban Varanasi [17, 31]. Overall, the results suggest that ML models based on nonclinical, survey‐based data can support low‐cost, scalable screening in resource‐limited rural settings. These tools facilitate targeted interventions, community‐specific prevention strategies and early detection of HTN risk.

Despite promising results, several limitations should be noted. First, the cross‐sectional design precludes causal inferences. Second, reliance on self‐reported behavioral data may introduce recall or social desirability bias. Third, the study′s geographic scope limited to one district may affect generalizability to rural or other regions of Bangladesh. Lastly, although the models performed well, future research should aim at improving robustness by including more diverse populations, clinical biomarkers, and exploring advanced algorithms. Finally, ML‐based models, particularly RF, offer a cost‐effective, noninvasive approach to early HTN risk prediction using easily collectable community‐level data. These tools can aid public health planning and clinical screening in resource‐limited settings, enabling timely and targeted interventions to reduce the growing burden of HTN in Bangladesh.

## 5. Conclusions

This study demonstrates the utility of ML approaches for predicting HTN and identifying its key risk factors among adults in the northern region of Bangladesh. With a prevalence of 36.5%, HTN represents a substantial public health burden in this population, exceeding national averages and highlighting potential regional disparities driven by socioeconomic and lifestyle factors. Among the 34 candidate predictors, 13 features including age, body weight, BMI, dietary habits, physical activity, education, family size, and income were consistently identified as significant contributors to HTN risk. Of the five ML models evaluated, the RF classifier exhibited superior predictive performance, achieving an accuracy of 72% and an AUC of 0.80. SHAP analysis further emphasized the relative importance of demographic, behavioral, and socioeconomic determinants, providing interpretable insights into modifiable risk factors. These findings underscore the potential of ML‐based frameworks to enhance early detection, inform targeted interventions, and optimize resource allocation for HTN prevention and management in rural Bangladesh. Future research should consider integrating longitudinal data and external validation cohorts to further refine predictive accuracy and generalizability across diverse populations.

## Author Contributions

All authors contributed significantly to the study. **Most. Nusrat Jahan Resma** contributed to study design, data collection and preprocessing, statistical analysis, and drafting of the manuscript. **Md. Kabir Bin Karim** was involved in literature review, data collection, statistical analysis, and result interpretation. **Isteaq Kabir Sifat** assisted with coding, visualization, validation of results, and manuscript editing. **Md. Abdul Kayum** contributed to data acquisition, software, and critical review of the manuscript. **Md. Kaderi Kibria** conceived and supervised the study, provided expertise in machine learning and biostatistics, guided interpretation, and finalized the manuscript.

## Funding

No funding was received for this manuscript.

## Ethics Statement

This study received ethical clearance from the Institutional Animal, Medical Ethics, Biosafety, and Biosecurity Committee (IAMEBBC) of the Institute of Biological Sciences, University of Rajshahi, Bangladesh (Approval No.: 209/320/(69)|AMEBBC/|BSc). All procedures were conducted in accordance with the ethical standards of the Declaration of Helsinki. Prior to participation, written informed consent was obtained from all respondents after providing a clear explanation of the study′s objectives, procedures, potential risks, and benefits. Confidentiality and anonymity were strictly maintained throughout the research process.

## Conflicts of Interest

The authors declare no conflicts of interest.

## Supporting Information

Additional supporting information can be found online in the Supporting Information section.

## Supporting information


**Supporting Information 1** Table S1: List of 34 covariates for hypertension (HTN) selected based on previous research. Table S2: Hyperparameter tuning of different classifiers using GridSearchCV. Table S3: Behavioral characteristics of study participants. Figure S1: Multistage sampling technique used to select participants from the population. Figure S2: Overview of the methodological framework and analytical steps. Figure S3: Receiver operating characteristic (ROC) curves of machine‐learning models for predicting HTN.

**Supporting Information 2** Appendix S1: Detailed description of the machine‐learning models applied in this study.

## Data Availability

The datasets analyzed in the current study are available from the corresponding author upon reasonable requests.
