# Supplementary material for: Machine‐Learning–Based Prediction of Hypertension and Its Risk Factors Among Adults in the Northern Region of Bangladesh
Source: J Diabetes Res. 2026 Mar 20;2026:1799434. doi: 10.1155/jdr/1799434 (PMC13140386; doi:10.1155/jdr/1799434)
Supplement: Supplementary file 1 — Supporting Information 1 Table S1: List of 34 covariates for hypertension (HTN) selected based on previous research. Table S2: Hyperparameter tuning of different classifiers using GridSearchCV. Table S3: Behavioral characteristics of study participants. Figure S1: Multistage sampling technique used to select participants from the population. Figure S2: Overview of the methodological framework and analytical steps. Figure S3: Receiver operating characteristic (ROC) curves of machine‐learning models for predicting HTN. [file JDR-2026-1799434-s001.docx]

Prediction of Hypertension and Its Risk Factors among Adults in Dinajpur District, Bangladesh: A Machine Learning Approach

**Supplementary File**

**Table S1:** List of 34 covariates of HTN selected based on a previous research article.

| **Variables** | **Data Type** | **Description** | **Categorization** |
| --- | --- | --- | --- |
| Residence | Binary | Permanent residential status of respondents | Rural  Urban |
| Gender | Binary | Respondent’s gender | Male  Female |
| Age | Continuous | Respondent’s age in years | -- |
| Marital Status | Ordinal | Respondent’s marital status | Single  Married  Divorced/Widowed |
| Education | Ordinal | Respondent’s education level | No Education  Primary Education  Secondary Education  Higher Secondary  Honours/Diploma or Above |
| Religion | Ordinal | Respondent’s religion | Muslim  Hindu  Buddhist |
| Family Income | Ordinal | Respondent’s family income | Less than 20000  20001-35000  35001-55000  55000-80000  More than 80000 |
| Family Size | Continuous | Number of family size | -- |
| Occupation | Nominal | Respondent’s Occupation | Farmer  Business  Day Labour  Driver  Employee  Housewife  Retired  Others  Unemployment |
| Owner of house | Binary | Housing ownership status of respondents | Personal  Rental |
| Loan | Binary | Respondent’s loan status | Yes  No |
| Ever Smoke Cigarette | Nominal | Respondent’s ever smoke cigarette or not | Yes  No |
| Betel leaf/gul | Binary | Respondent’s eat betel leaf or not | Yes  No |
| Tea/Coffee | Binary | Respondent’s drink tea or coffee or not | Yes  No |
| Eat Fruit | Ordinal | Weekly frequency of fruit consumption by respondent | 1-2 days  3-4 days  5-7 days  None |
| Eat Vegetable | Ordinal | Weekly frequency of vegetable consumption by respondent | 1-3 days  4-5 days  6-7 days  None |
| Eat Meat | Ordinal | Weekly frequency of meat consumption by respondent | 1-2 days  3-5 days  6-7 days  None |
| Eat Sweet | Nominal | Whether the respondent eats sweet weekly or not | Yes  No |
| Add Salt | Binary | Respondent’s added extra salt on food or not | Yes  No |
| Vigorous Activity | Nominal | Whether the respondent engages in vigorous activity per week or not | Yes  No |
| Sports | Binary | Weekly sports participation of respondents | Yes  No |
| Walk | Binary | Weekly walking status of respondents | Yes  No |
| Travel | Ordinal | Mode of travel to other places among respondents | Foot  Bi-cycle  Engine Vehicle |
| Measure of HTN | Binary | Respondent’s measure HTN or not | Yes  No |
| HTN | Binary | Hypertension status of respondents |  |
| History of HTN | Binary | Respondent’s family history regarding hypertension | Yes  No |
| Diabetes | Binary | Diabetes status of respondents | Yes  No |
| History of DBT | Binary | Respondent’s family history regarding Diabetes | Yes  No |
| Kidney Disease | Binary | Kidney disease status of respondents | Yes  No |
| CVD | Binary | Cardiovascular Disease status of respondents | Yes  No |
| History of CVD | Binary | Respondent’s family history regarding CVD | Yes  No |
| Height(inch) | Continuous | Respondent’s height in inch | -- |
| Weight(kg) | Continuous | Respondent’s weight in kg | -- |
| BMI | Ordinal | Body Mass Index Level of respondents | Normal  Overweight  Obese |

**Table S2:** Hyper parameters tuning of different classifiers using GridSearchCV

| **Classifier** | **Hyper-parameter and value** |
| --- | --- |
| LR | C= **1.0**, penalty= l2, solver= liblinear, max_iter=10 |
| DT | criterion=gini, max_depth=20, min_samples_split=10, min_samples_leaf=5, max_features = None |
| RF | n_estimators=100, max_features=sqrt, max_depth=20, min_samples_split=4, min_samples_leaf=1 |
| XGBoost | n_estimators=100, max_depth=6, learning_rate=0.1, subsample=0.9, colsample_bytree=1.0 |
| LightGBM | num_leaves=5, learning_rate=.1, n_estimators=100 |

**Table S3:** Behavioral characteristics of study participants.

| **Variable** | **Categories** | **Frequency (%)** |  | **Variable** | **Categories** | **Frequency (%)** |
| --- | --- | --- | --- | --- | --- | --- |
| Ever smoke cigarette | Yes | 207 (20.2%) |  | Eat meat (days per week) | 1-2 days | 715 (69.7%) |
|  | No | 819 (79.8%) |  |  | 3-5 days | 211 (20.6%) |
| Eat Betel/Gul | Yes | 321 (31.3%) |  |  | 6-7 days | 38 (3.7%) |
|  | No | 705 (68.7%) |  |  | None | 62 (6.0%) |
| Drink Tea/Coffee | Yes | 595 (58.0%) |  | Eat fruit (days per week) | 1-2 days | 551 (53.7%) |
|  | No | 431 (42.0%) |  |  | 3-4 days | 247 (24.1%) |
| Eat sweet | Yes | 540 (52.6%) |  |  | 5-7 days | 120 (11.7%) |
|  | No | 486 (47.4%) |  |  | None | 108 (10.5%) |
| Usual mode of transport | On foot | 218 (21.3%) |  | Eat vegetable (days per week) | 1-3 days | 23 (2.2%) |
|  | Bicycle | 70 (6.8%) |  |  | 4-5 days | 150 (14.6%) |
|  | Engine Vehicle | 738 (71.9%) |  |  | 6-7 days | 851 (82.9%) |
| Use top added salt | Yes | 313 (30.5%) |  |  | None | 2 (0.2%) |
|  | No | 713 (69.5%) |  |  | | |


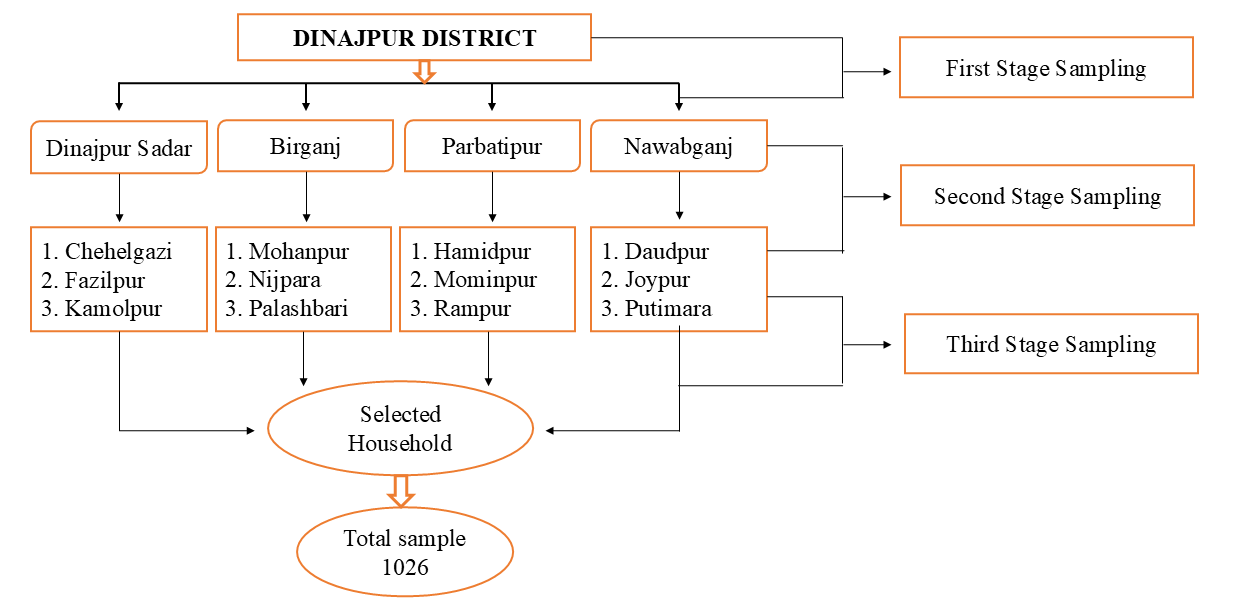
**Figure S1:** Muti-stage sampling technique to draw samples from population.


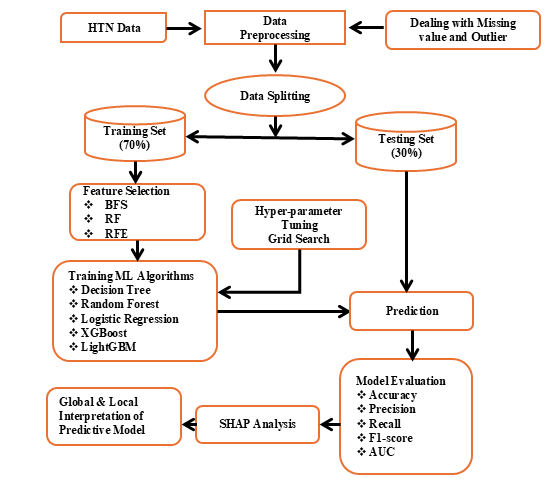


**Figure S2:** Overview of the methodological framework and analytical steps.


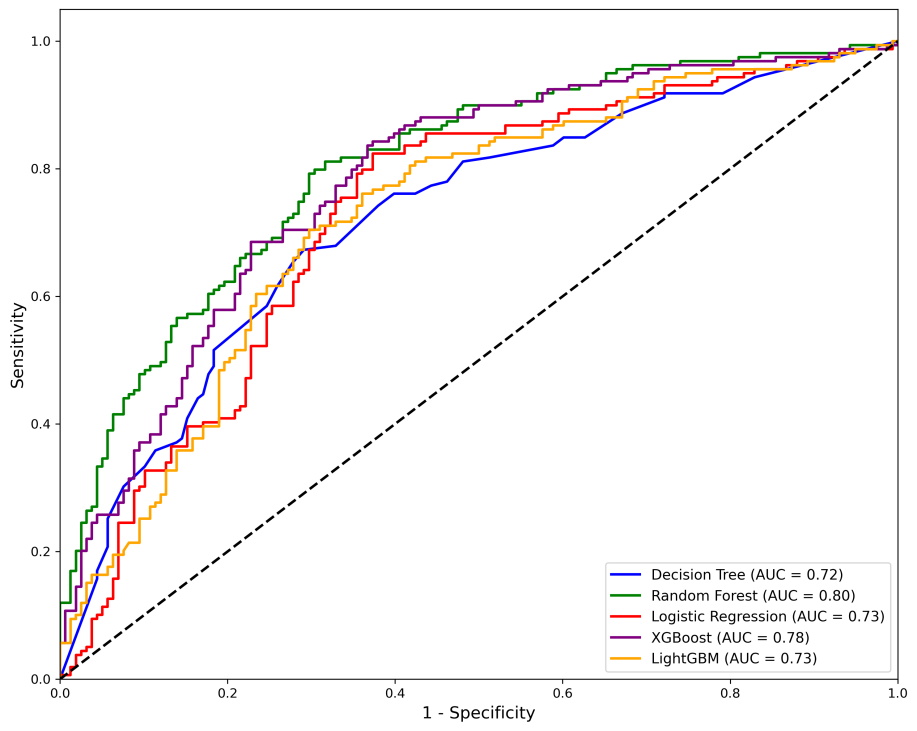


**Figure S3:** ROC curves of machine leaning models for predicting HTN.
