# Supplementary material for: Machine‐Learning–Based Prediction of Hypertension and Its Risk Factors Among Adults in the Northern Region of Bangladesh
Source: J Diabetes Res. 2026 Mar 20;2026:1799434. doi: 10.1155/jdr/1799434 (PMC13140386; doi:10.1155/jdr/1799434)
Supplement: Supplementary file 2 — Supporting Information 2 Appendix S1: Detailed description of the machine‐learning models applied in this study. [file JDR-2026-1799434-s002.docx]

**Machine Learning Algorithms**

**Logistic regression (LR)**

Logistic regression (LR) is the most widely used supervised ML algorithm that uses probability. The most well-liked supervised machine learning algorithm, LR, is mostly applied to classification tasks [33]. The likelihood of the response (HTN as well as non-HTN) is predicted by this model using the logistic function in response to different input feature values. The following is a representation of the logistic function

$Logit\left( p_{j} \right)=log\left( \frac{p_{j}}{1-p_{j}} \right)=\beta_{0}+\beta_{1}x_{1j}+\beta_{2}x_{2j}+...+\beta_{k}x_{kj}+\epsilon_{j}, j=1,2,...,n . . . . . . (i)\text{ }$

Where,$p_{j}$denote the probability that  *j^th^* person will have HTN;$X_{kj}$is the *k^th^* input feature of the *j^th^* individual and$\beta_{k}$is the *k^th^* regression coefficients.

The above equation (i) can be written as

$P=\frac{exp\left( \beta_{0}+\beta_{1}+\beta_{2}+...+\beta_{k}x_{kj} \right)}{1+exp\left( \beta_{0}+\beta_{1}+\beta_{2}+...+\beta_{k}x_{kj} \right)}\text{ }$

and odds as

$\frac{p}{1-p}=exp\left( \beta_{0}+\beta_{1}+\beta_{2}+...+\beta_{k}x_{kj} \right)\text{ }$

If $\frac{p}{1-p}>1,$therefore we categorize as HTN, while $\frac{p}{1-p}<1,$therefore we categorize as non-HTN.

**Decision Tree (DT)**

A DT Classifier is a popular machine learning algorithm used for both classification and regression tasks. In the context of classification, it works by recursively partitioning the input space into regions and assigning a class label to each region. [34] These partitions are represented in a tree-like structure, where each internal node represents a feature (or attribute), each branch represents a decision based on that feature, and each leaf node represents the outcome or the class label. A decision tree algorithm constructs a tree-like model for decision-making by recursively partitioning the data based on feature values. Here's a succinct description of the decision tree algorithm:

1. Start with the Entire Dataset: Begin with the root node representing the entire dataset.
2. Select the Best Split: At each node, evaluate all possible splits for all features. Use a criterion such as Gini impurity, entropy (information gain), or variance reduction to determine the best split that maximizes the separation of the classes (for classification) or minimizes the variance (for regression).

- Gini Impurity: $Gini\left( D \right)=1-\sum_{i=1}^{c} P_{i}^{2}$
- Entropy : $Entropy\left( D \right)= -\sum_{i=1}^{c} p_{i}{log}_{2}(p_{i})$

1. Split the Data: Divide the dataset into subsets based on the best split.
2. Create Child Nodes: For each subset created, generate child nodes.
3. Repeat Recursively: Apply steps 2-4 recursively to each child node using their respective subsets, continuing until one of the stopping conditions is met (e.g., maximum depth, minimum number of samples per leaf, or no further information gain).
4. Assign Terminal Nodes: Once the stopping condition is met, assign a class label (for classification) or a mean value (for regression) to the terminal (leaf) nodes.

The resulting tree structure can then be used to make predictions by traversing from the root node to a leaf node, following the splits that correspond to the feature values of the input data.

**Random Forest (RF)**

RF is an ensemble machine learning method that uses decision trees as its base classifier[35]. RF were chosen for their ability to improve decision trees by using bagging to build multiple trees, addressing overfitting through averaging predictions [21].Every decision tree is constructed using a variety of features and sub-datasets This approach helps to reduce the variance of the DT [36]. The steps in the RF algorithm are as follows

- **Step 1:** Each decision tree in the Random Forest model is built using a subset of features and a subset of data points. To put it simply, *m* features and *n* random records are selected from a data set containing k records
- **Step 2:** For every sample, a distinct decision tree is built.
- **Step 3:** Each DT produces an output.
- **Step 4:** In CRT, the final output is determined by either averaging or by majority voting.

**Extreme gradient boosting (XGB)**

XGBoost is a portable, adaptable, and highly efficient tool for solving regression and classification problems [37]. Boosting, a learning approach used by XGBoost, aims to create a strong classifier from weaker learners or classifiers. Both weak and strong classification models indicate the relationship between the predicted and actual classes. By iteratively stacking classifiers, each subsequent classifier corrects the errors of the previous one. This process is repeated until the training dataset accurately predicts the target variable's class label [20]. The tree-derived predictions can be expressed mathematically as follows

- $\hat{y}=\phi\left( x \right)=\frac{1}{n}\sum_{k=1}^{n} f_{k}\left( x \right)$

where 𝑌̂ represents the predicted 𝐸𝑇𝑜 , with 1 ≤ 𝑘 ≤ 𝑛 and 𝑛 being the total number of functions learned by the 𝑛 trees. The model minimizes the following regularized objective L(𝜙) to learn the set of functions 𝑓_k_ used in the model:

- $L\left( \phi\right)=\sum_{i} l\left( \hat{y_{i}},y_{i} \right)+\sum_{k} \Omega\left( f_{K} \right)$
- Where $\Omega\left( f_{k} \right)=\gamma T+1/2\lambda\|w{\|}^{2}$

where the difference between ̂𝑦_i_ (prediction) and 𝑦_i_ (target) is measured by the differentiable convex loss function, 𝑙. 𝛺 is an additional regularization term that discourages the growth of additional trees in the model in order to avoid complexity and hence lessen overfitting. A tree's number of leaves T, its complexity, a penalty parameter, and the vector of scores on its leaves are denoted by the variables respectively 𝛾, 𝜆, and ‖𝑤‖ [38].

**Light gradient boosting machine (LGBM)**

The advanced LGBM algorithm has been utilized for HTN prediction. LGBM employs tree-based learning algorithms within a gradient boosting framework, optimized for distribution and efficiency through Gradient-based One-Side Sampling (GOSS) and Exclusive Feature Bundling (EFB) techniques [39]. In LGBM, multiple tree models are trained additively, where each new tree model learns to predict the residuals (errors) of the previous models [40]. Unlike XGBoost, LGBM utilizes histogram-based algorithms to speed up training and lower memory consumption. It also employs a leaf-wise growth strategy with depth constraints. The histogram algorithm converts continuous floating-point values into *k* bins and constructs a histogram with a width of *k* [41]. The LGBM algorithm, based on decision tress, is formulated as follows: Given a training data set $S=\left( x_{i},y_{i} \right);i=1, 2,\cdot\cdot\cdot,n;x_{i}\in R^{m},y_{i}\in R$ where *n* is the sample and *m* is the number of features. To obtain the estimation, the predictions of the decision trees are combined as follows:

$$\hat{y}_{i}^{LG}=\sum_{p=1}^{p} f_{p}\left( xi \right)$$

where there are *p* tress with *f_p_* trees. To obtain *f_p_* the objective is to minimize the objective function below

$$f_{p}=arg\min_{f_{p}}\sum_{i=1}^{1} L\left( y_{i},{ŷ}_{i}^{LG\left( p \right)} \right)+\Omega\left( f_{p} \right)$$

The regularization parameter **Ω** and the loss function is *L* which is given by

$$\Omega\left( f_{p} \right)=\alpha T+\frac{1}{2}\lambda\sum_{j=1}^{T} w_{j}^{2}$$

Where *α* and *λ* represent the penalty parameters for *T* leaves and the weight of leaves *w*. Taking *L* as a loss function which is a squared error

${L\left( y_{i},\hat{y}_{i}^{LG\left( p-1 \right)}+f_{p}\left( x \right) \right)=\left( y_{i}-\hat{y}_{i}^{LG\left( p-1 \right)}-f_{p}\left( x \right) \right)}^{2}\left( r-f_{p}\left( x \right) \right)^{2}$

Then the residual *r* is adjusted to obtain *f_p_*. A quadratic approximation is used to define the function for minimizing the objective function at iteration *p* as

$f_{p}\simeq arg{\min_{f}}_{p}\sum_{i=1}^{n} \left[ g_{i} | f_{p}\left( x_{i} \right)+1/2h_{i}f_{i}^{2}\left( x_{i} \right) \right]+\Omega\left( f_{p} \right)$,

$g_{i}=\partial_{\hat{y}_{i}^{LG\left( p-1 \right)}}L\left( y_{i},{ŷ}_{i}^{LG\left( p-1 \right)} \right)$ ,

$h_{i}=\partial_{\hat{y}_{i}^{LG\left( p-1 \right)}}^{2}L\left( y_{i},{ŷ}_{i}^{LG\left( p-1 \right)} \right)$,

By minimizing the objective function, a new tree *f_p_* is derived. Each node is split by the decision tree based on the maximum information gain. The variance gain for a node that splits feature *j* at point *s* is given by

$$Z_{j\mid0}(s)=\frac{1}{n_{0}}\left\{ \frac{\left( \sum_{\left\{ x\in\in O:x_{i}\leq s \right\}} g_{i} \right)^{2}}{n_{l|0|}^{j}(s)}+\frac{\left( \sum_{\left\{ x_{i}\in O:x_{j}>s \right.} g_{i} \right)^{2}}{n_{r|0|}^{j}(s)} \right\},$$

O is samples on the decision tree fixed node $n_{o}=\sum I\left[ x_{i}\in O \right]$,

$n_{l/o}^{j}\left( s \right)=\sum I\left[ x_{i}\in O:x_{ij}\leq s \right]$ and $n_{l/o}^{j}\left( s \right)=\sum I\left[ x_{i}\in O:x_{ij}\leq s \right]$. The decision tree selects$s_{j}*arg\max_{s}z_{j}\left( s \right)$ for each feature *j* and calculates the highest gain Z_i_(s_j_^*^). Based on feature j* at point s_j_*, the data will be divided into the left and right nodes. To determine the best splitting point and compute the information gain, all samples are scanned.
